# Changes in Oxidative Stress and Inflammatory Biomarkers in Fragile Adults over Fifty Years of Age and in Elderly People Exclusively Fed Enteral Nutrition

**DOI:** 10.1155/2016/5709312

**Published:** 2015-12-01

**Authors:** Maria D. Mesa, Josune Olza, Carolina Gonzalez-Anton, Concepcion M. Aguilera, Rosario Moreno-Torres, Africa Jimenez, Antonio Perez de la Cruz, Azahara I. Ruperez, Angel Gil

**Affiliations:** ^1^Department of Biochemistry and Molecular Biology II, Institute of Nutrition and Food Technology “Jose Mataix”, Biomedical Research Centre, Health Science Technological Park, University of Granada, Avenida del Conocimiento s/n, 18016 Granada, Spain; ^2^Clinical Nutrition and Dietetic Unit, University Hospital Virgen de las Nieves, Avenida de las Fuerzas Armadas 2, 18014 Granada, Spain; ^3^Vegenat, S.A. Research and Development Department, Ctra Badajoz-Montijo, km 24,9, Pueblonuevo del Guadiana, 06184 Badajoz, Spain

## Abstract

We aim to evaluate whether exclusive feeding of an enteral formula enriched with *n*-3 long chain polyunsaturated fatty acids (*n*-3 LC-PUFA) affects oxidative stress and the antioxidant defence system and may improve the levels of some relevant inflammatory, and cardiovascular biomarkers in frail adults over fifty years of age and in elderly subjects. Fifty-five patients were divided into two groups and were exclusively fed a newly designed normoproteic and isocaloric enteral formula enriched with eicosapentaenoic (98 mg/d) and docosahexaenoic acids (46 mg/d) (*n* = 26) or a reference enteral diet (*n* = 29). Oxidative, inflammatory and cardiovascular risk biomarkers and red blood cell fatty acid profiles were determined at the beginning and after 90 and 180 days of feeding. The *n*-3 LC-PUFA percentage tended to be higher (*P* = 0.053) in the experimental group than in the reference group. Administration of the *n*-3 LC-PUFA diet did not increase oxidative stress or modify plasma antioxidant capacity but decreased antioxidant enzymatic activities. MMP-9 plasma concentration decreased with both formulae, whereas tPAI-1 tended to decrease (*P* = 0.116) with the administration of the experimental formula. In conclusion, administration of the new *n*-3 LC-PUFA-enriched product for 6 months did not negatively alter the oxidative status and improved some cardiovascular risk biomarkers.

## 1. Introduction

Active ageing is becoming an important topic for the process of optimizing opportunities for health, participation, and security to improve the quality of life of aged people. Lifestyle changes, including physical activity and a healthy suitable diet, are the most important strategies available to modulate health outcomes [1, 2]. Ageing is a physiological process characterized by a number of changes in the body and which impair the ability to respond to stress, which increases the risk of diseases such as cardiovascular disease (CVD) and other chronic diseases [3, 4]. Accumulating evidence suggests that age-related diseases are associated with increased oxidative stress [5, 6], an impaired antioxidant defence system [7], and deregulated immune and inflammatory responses [3, 7, 8]. Moreover, silent inflammation during ageing is evidenced by increased circulating levels of proinflammatory cytokines, acute phase proteins, and organ dysfunction that leads to the development of several diseases [3]. In addition, the close association between risk of malnutrition and functional impairment requires nutritional intervention strategies to prevent the development and progression of several diseases [3]. Fragile adults affected by severe chronic diseases also exhibit an altered oxidative status [9] and a derangement in the antioxidant defence system [10].

Enteral nutrition (EN) containing specific nutrients and bioactive compounds are used in a number of situations when oral feeding is not an option, particularly in fragile, aged patients [11, 12]. In general, enteral formulae contain different lipid mixtures and represent a reliable supply of energy and essential fatty acids. Lipid emulsions made from soybean oil in various combinations with medium-chain triacylglycerols (TAG), olive oil, and fish oil are widely used according to the purpose of the formula [13]. One of these emulsions includes the use of purified* n*-3 long chain polyunsaturated fatty acids (*n*-3 LC-PUFAs), that is, eicosapentaenoic acids (EPA), and docosahexaenoic acids (DHA) [14]. These fatty acids play an important role in modulating the inflammatory response and alleviating symptoms in patients at high risk of developing inflammatory diseases [15–18]. Recently, two systematic reviews demonstrated that dietary* n*-3 fatty acids are associated with lower levels of inflammation and endothelial activation plasma biomarkers in patients with CVD and other chronic and acute diseases [19]. Moreover, dietary* n*-3 PUFAs are associated with improved systemic insulin sensitivity [20]. An appropriate balance between* n*-3/*n*-6 PUFAs in the diet diminishes the risk of CVD due to the anti-inflammatory properties of* n*-3 PUFAs [21] and the proinflammatory character of* n*-6 PUFAS such as *γ*-linolenic acid and dihomo-*γ*-linolenic acid, upregulated in the modulation of the inflammatory response [22]. However, once incorporated into cell membranes,* n*-3 LC-PUFAs are more susceptible to oxidation [23], which could trigger oxidative stress-derived alterations, leading to increased inflammatory processes [4]. Therefore, more evidence is needed to establish dietary recommendations for these fatty acids in the elderly [19].

Our research group previously observed that feeding exclusively with an EPA and DHA-enriched enteral formula allowed* n*-3 LC-PUFA incorporation into total plasma lipids (specifically EPA in the phospholipids and cholesterol ester fractions) and normalized plasma TAG levels after 3 months of feeding [24]. However, the potential benefits of dietary* n*-3 LC-PUFA administration on oxidative stress and inflammatory and cardiovascular risk biomarkers using that enteral formula have not been previously addressed. Therefore, the present study determined the influence of that enteral formula enriched in* n*-3 LC-PUFA on oxidative, inflammatory, and cardiovascular risk biomarkers and the impact on the antioxidant defence system following exclusive enteral feeding for 6 months in fragile adults aged over fifty years and in elderly patients.

## 2. Methods

### 2.1. Subject Selection and Allocation

Fifty-five outpatients (mean age 79 ± 1 years and range 52 to 97 years; *n* > 50 < 65 = 6 and *n* > 65 = 49) from the Unit of Clinical Nutrition at the University Hospital Virgen de las Nieves (Granada) were recruited and asked to participate in the study. The inclusion criteria were the prescription of total EN for at least 6 months and voluntary consent for participation. The exclusion criteria were an unstable clinical situation, fatal illness, refusal to participate in the study, or being enrolled in another clinical trial. Initially, we selected fifty-five outpatients. Concomitant diseases were mainly cognitive deficits and Alzheimer's disease, cerebrovascular diseases and cardiovascular events, and cancer in few occasions. Medications used by these patients were mainly gastric protectors, psychodrugs, anticoagulants and antihypertension medication, and, with less frequency, diuretics, analgesics, antiarrhythmics, and antidiabetic drugs in few occasions. The patients were randomly allocated into two groups: experimental (*n* = 26) and reference (*n* = 29). In the experimental group, five patients withdrew, four died, two changed their diets, and one no longer required EN. In the reference group, five patients withdrew, three died, and two changed their diets. Thus, at the end of the intervention, thirty outpatients (age 79 ± 2 years; range 52 to 94 years) (experimental group (*n* = 14) and reference group (*n* = 19)) completed this study. Mean baseline tricipital skinfold was 17.6 mm and 17.5 mm for the experimental and the reference groups, respectively, while midarm circumference was 24 cm and 25 cm for the experimental and the reference groups, respectively.

The study was approved by the Ethics Committee of the University Hospital Virgen de las Nieves from Granada. All procedures were performed in accordance with the institutional guidelines following the IHC Harmonized Tripartite Guideline for Good Clinical Practice in accordance with the* Helsinki Declaration of the World Medical Association: Ethical Principles for Medical Research on Human Beings* (revised in Edinburgh, October 2000). All individuals or subjects' caregivers provided written informed consent prior to their inclusion in the study.

### 2.2. Study Design and Performance

The present study was a randomized, experimental, prospective, and intention*-*to-treat study (6-month duration) with two parallel groups that were fed exclusively with EN. In the experimental group, a newly designed normoproteic (40 g/100 mL) and isocaloric enteral formula (T-Diet Plus, Vegenat S. A., Badajoz, Spain) containing 75 mg/L EPA and 35 mg/L DHA was administered. In the reference group, a standard normoproteic (4 g/100 mL) and isocaloric enteral formula (Jevity, Abbot Laboratories, USA) (without* n*-3 LC-PUFAs) was administered.  Table 1 presents the nutritional composition of the experimental and reference diets.

The subjects were provided 1500 mL of the diet, which guaranteed their daily energetic and nutritional requirements. The overall mean daily intake was 1266 ± 60 mL/d (mean ± SEM) for the T-Diet Plus group and 1362 ± 50 mL/d for the reference group. There was no difference in the daily intake between the groups. The mean EPA and DHA intake of subjects receiving the T-Diet Plus was approximately 94 and 44 mg/d, respectively. Administration of both products was performed as a bolus using a nasogastric feeding tube with a large-bore syringe or via an ostomy. To maintain the optimal hydration state, all patients received 1000–1200 mL of water daily.

### 2.3. Blood Samples

Fasting blood samples were collected between 8:00 and 10:00 am at time 0 and after 90 and 180 days. The serum and plasma (EDTA-coated tubes) were separated by centrifugation (15 min at 1750 ×g) and were immediately frozen at −80°C. The buffy coat was mixed with RPMI (1 : 1), layered onto Histopaque, and centrifuged to obtain lymphocyte isolation (700 ×g, 30 min at 20°C). Once isolated, the lymphocytes were washed with RPMI 10% FBS and were frozen gradually at −80°C in FBS 10% DMSO until use. Finally, the red blood cells (RBCs) were washed three times with NaCl 0.9%, lysed with cold water, and frozen at −80°C until use.

#### 2.3.1. Plasma Lipids

Plasma TAG, total cholesterol (TC), low-density lipoprotein cholesterol (LDLc), apolipoprotein B (apoB), high-density lipoprotein cholesterol (HDLc), and apolipoprotein A-I (apoA-I) concentrations were determined by standardised spectrophotometric techniques using a Roche Hitachi Modular DDP clinical analyser system (Roche Diagnostics España, S. L., Barcelona). All parameters were analysed at the laboratory of the Virgen de las Nieves University Hospital (Granada).

#### 2.3.2. Oxidative Stress Biomarkers

An enzymatic immunoassay for the quantitative determination of plasma oxidized low-density lipoprotein (oxLDL) was used (Cat. number BI-20042, Biomedica Medizinprodukte GmbH & Co KG, Vienna, Austria). After LDL isolation [25], LDL oxidation was induced by incubating with 20 *μ*M CuSO_4_. LDL susceptibility to oxidation was determined as previously described [26]. Conjugated dienes were measured after 300 min, and the amount of lipid hydroperoxides was determined after 4 h of incubation. Peripheral blood lymphocyte DNA damage was determined using the double-strand break analysis (termed the COMET assay) as previously described [27]. Briefly, frozen lymphocytes were lysed, and the cellular proteins were removed. DNA electrophoresis was performed to allow for separation of the two DNA strands. 6-diamidino-2-phenylindole (DAPI)-stained nucleotides were examined with a UV microscope with an excitation filter of 435 nm and at 400x magnification.

#### 2.3.3. Nonenzymatic Antioxidant Defence System

Plasma total antioxidant capacity (TAC) was assessed using a spectrophotometric commercial assay kit (Cat. number 709001, Cayman, MI, USA). After extraction with 1-propanol, the plasma concentrations of retinol, tocopherol, and coenzyme Q_10_ (CoQ_10_) (oxidized, reduced, and total) were determined by high pressure liquid chromatography coupled to an electrochemical detector (HPLC-EC) according to Battino et al. [28]. The amount of beta-carotene was also determined after extraction with 1-propanol in an HPLC system attached to a multiwavelength ultraviolet detector set at 450 nm. All compounds were identified by predetermining the retention times of individual standards.

#### 2.3.4. Enzymatic Antioxidant Defence System

The haemoglobin (Hb) concentration in the blood samples was determined spectrophotometrically by the colorimetric cyanmethemoglobin method [29] using Sigma Diagnostic reagents. RBC antioxidant enzyme activities were determined spectrophotometrically as described previously with slight modifications for microplate analysis. RBC catalase activity was assayed as described by Aebi [30] and was expressed as nmol/(L·gHb). RBC superoxide dismutase (SOD) activity was assayed according to the methods of McCord and Fridovich [31] and was expressed as U/mg Hb. RBC glutathione reductase (GR) activity was assayed by the method of Carlberg and Mannervik [32] and was expressed as U/g Hb. RBC glutathione peroxidase (GPx) activity was assayed by the coupled enzyme procedure with tert-butyl hydroperoxide as a substrate [33] and was expressed as U/g Hb.

#### 2.3.5. Inflammatory and Cardiovascular Risk Biomarkers

MILLIPLEX kits (Linco Research, MO, US) were used with a Luminex 200 System (Luminex Corporation, TX) to determine the amount of soluble intercellular adhesion molecule- (sICAM-) 1 (CV: 7.9%), soluble vascular cell adhesion molecule- (sVCAM-) 1 (CV: 4.5%), soluble endothelial selectin (sE-selectin) (CV: 11.2%), matrix metalloproteinase- (MMP-) 9 (CV: 6.8%), myeloperoxidase (MPO) (CV: 12.3%), total plasminogen activator inhibitor- (tPAI-) 1 (CV: 6.6%) (Cat. HCVD1-67AK), interleukin- (IL-) 6 (CV: 7.8%), tumour necrosis factor- (TNF-) *α* (CV: 7.8%), and monocyte chemotactic protein (MCP)-1 (CV: 7.9%) (Cat. HADK2-61 K-B). Endothelin-1 was analysed by ELISA (Cat. number BI-20052, Biomedica Medizinprodukte GmbH & Co KG, Vienna, Austria). High sensitive C reactive protein (hsCRP) was quantified with a turbidimetric assay (Dade Behring Inc., Deerfield, IL).

#### 2.3.6. Quantification of Fatty Acids in Red Blood Cells

RBC lipids were dissolved with isopropanol (25 mg/L BHT) and were extracted with hexane 3 times. The hexane phase was evaporated, and the fatty acids were identified and quantified after methylation by gas-liquid chromatography using a 60 m long capillary column (32 mm internal diameter and 20 mm film thickness) impregnated with SP 2330 FS (Supelco, Bellefonte, CA, USA). Fatty acid methyl esters from plasma lipids were obtained as previously reported [34]. Briefly, the hexane extracts of the total plasma and lipid fractions were dissolved into 2 mL methanol : benzene (4 : 1 v/v). Methylation was performed at 100°C for 1 h by adding 200 *μ*L acetyl chloride. After cooling, 5 mL of 0.43 M K_2_CO_3_ was added to stop the reaction and neutralize the mixture. The tubes were then shaken and centrifuged. The benzene upper phase was dried under N_2_ and was resuspended with 100 *μ*L hexane.

### 2.4. Statistical Analysis

All data are presented as the mean ± SEM. Prior to statistical analyses, all variables were checked for normality and homogeneous variance using the Kolmogorov-Smirnov and Levene tests, respectively. Variables that did not follow normality were logarithmically (apoA-I, dienes slope, TAC, tocopherol, total CoQ_10_, MMP-9, sVCAM-1, eSelectin, tPAI-1, IL-6, and hsCRP) or inversely (HDLc, sICAM-1, and GR) transformed. To test for differences between the groups at baseline and each time point, a *t*-test for independent data was performed on the variables (or transformed variables) following a normal distribution for each group. A nonparametric Mann-Whitney* U* test was performed for variables that did not follow a normal pattern (apoB, oxLDL, COMET tail moment, catalase, SOD, MCP-1, endothelin-1, TNF-*α*, and MPO). For the data with a normal distribution and within each group, a one-way ANOVA and* a posteriori* Bonferroni tests were performed to evaluate the differences between feeding times. When the variables or transformed variables did not follow a normal pattern, Kruskal-Wallis and* a posteriori* Mann-Whitney* U* tests were performed. To establish differences between groups and the interaction time × group, we used a general linear model of variance for repeated measures (GLM-RM) for patients who completed the study (*n* = 14 and *n* = 19 for the experimental and reference groups, resp.). A value of *P* < 0.05 was considered significant. The data analyses were performed using a statistical software package (SPSS for Windows, 15.0, 2005, SPSS Inc., Chicago, IL, USA).

## 3. Results

During the study period, the haematological parameters were controlled in all participants. No changes in white blood cells, coagulation indicators, or plasma electrolytes were observed (data not shown).

### 3.1. Plasma Lipids

Plasma concentrations of TAG, TC, apoB, HDLc, LDLc, and apoA-I were similar between both groups of intervention at all times. Feeding the two diets did not significantly modify TC, LDLc, apoB, HDLc, or apoA-I, but the effect of each diet tended to be different for LDLc (*P* = 0.088) and HDLc (*P* = 0.069) (Table 2). In addition, the intervention significantly affected plasma TAG (*P* = 0.042), especially in the experimental group, since plasma concentrations decreased after the intervention with the experimental diet but not after the reference diet (*P* value for time per group interaction 0.074) (Table 2).

### 3.2. Oxidative Stress Biomarkers

When analysing diene formation during* in vitro*-induced LDL oxidation, the lag phase decreased after 90 days of feeding the experimental diet but returned to baseline conditions after 180 days. The lag phase was constant in subjects administered the reference product. However, the rate of* in vitro*-induced diene formation increased with time only in the reference group. LDL* in vitro*-induced hydroperoxide levels were lower in the experimental* versus* the reference group and this value did not change with dietary feeding in either group (Table 3). The oxLDL plasma concentrations were similar during the intervention in both groups (Table 3). There were no differences regarding lymphocyte DNA damage due to dietary intervention in either group, although the Comet Tail Moment was higher in the experimental group at baseline and throughout the interventional period (Table 3).

### 3.3. Antioxidant Defence System

**Figure 1:**
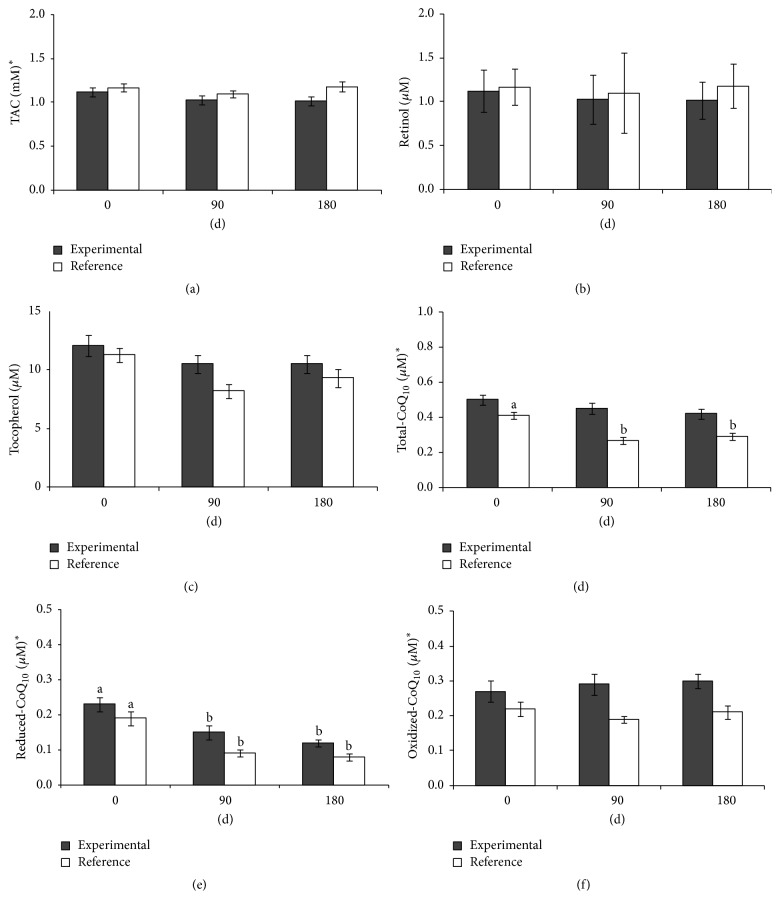
Plasma nonenzymatic antioxidant defence system parameters (a) TAC, (b) retinol, (c) tocopherol, (d) total-CoQ_10_, (e) reduced-CoQ_10_, and (f) oxidized-CoQ_10_ of frail adults and elderly patients fed exclusively by total enteral nutrition with the experimental and reference diets for 90 d and 180 d. ^*∗*^Significant differences between groups according to a general lineal model for repeated measures. Different letters indicate significant differences between times within each group. *P* < 0.05 was considered significant. TAC, total antioxidant capacity.

**Figure 2:**
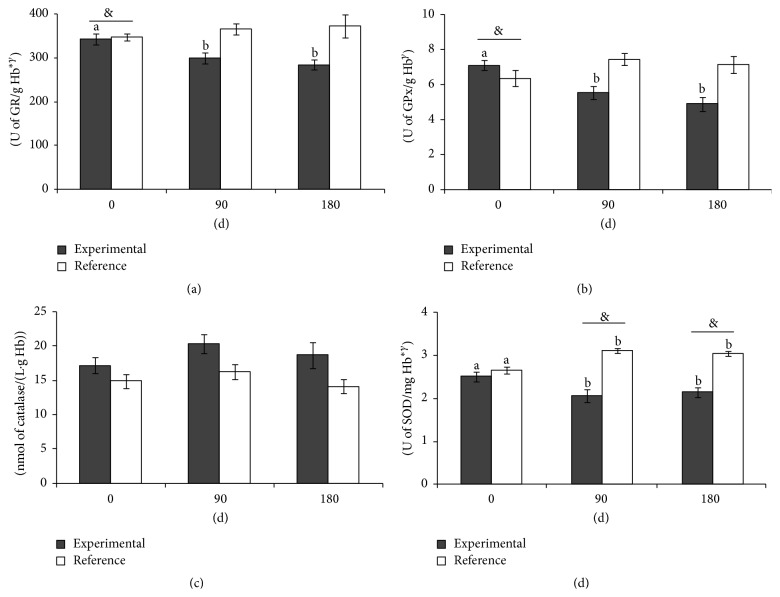
Red blood cell enzymatic antioxidant activities (a) GR, (b) GPx, (c) catalase, and (d) SOD of frail adult and elderly patients fed exclusively by total enteral nutrition with the experimental and reference diets for 90 d and 180 d. ^*∗*^Significant differences between groups according to GLM-RM. ^&^Significant differences between groups at each time point. ^*γ*^Significant differences in the interaction time × group according to GLM-RM. Different letters indicate significant differences between times within each group. *P* < 0.05 was considered significant. GLM-RM, general lineal model for repeated measures; GPx, glutathione peroxidase; GR, glutathione reductase; SOD, superoxide dismutase.

Plasma TAC and retinol concentrations were unmodified by the two diets. However, the TAC values were lower in the experimental group (Figure 1). Plasma tocopherol levels had decreased in the reference group at 90 days but recovered to baseline levels after 180 days. The values did not change with the experimental diet. CoQ_10_, both oxidized and reduced, were lower in the experimental group. The effect of enteral feeding was similar in both groups. Total-CoQ_10_ and reduced-CoQ_10_ were decreased at 90 and 180 days in the reference group. Only reduced-CoQ_10_ significantly decreased in the experimental group (Figure 1). Antioxidant GR, catalase, and SOD enzymatic activities were different between groups, and the effects of both enteral diets were significantly different for GPx, GR, and SOD (time × treatment effect). GPx, GR, and SOD activities decreased after administration of the experimental formula. Catalase activity did not change. GPx, GR, and catalase activities were unmodified in the reference group. SOD was increased at 90 and 180 days in the reference group (Figure 2).

### 3.4. Inflammatory and Cardiovascular Risk Biomarkers

The plasma levels of inflammatory and CVD risk biomarkers are presented in Table 4. The levels of inflammatory biomarkers (TNF-*α*, IL-6, and hsCRP) were similar in all volunteers at the beginning of the study and at 90 and 180 days. Regarding vascular function, endothelin-1 was lower in the experimental group at 90 and 180 days than in the reference group. Administration of both the experimental and reference formulae for 90 and 180 days significantly decreased the plasma concentrations of MMP-9. The effect on tPAI-1 was different between diets (time × treatment) (Table 4). tPAI-1 tended to decrease over time (*P* = 0.116) only in the experimental group.

### 3.5. Fatty Acids in Red Blood Cell Membranes

Table 5 presents the fatty acid profiles of RBC membranes. The linoleic acid (LA) and linolenic acid (LNA) percentages were different in both groups. LA was higher at 90 and 180 days, whereas LNA was lower at baseline and 90 days in the experimental than in the reference group. In addition,* n*-3 LC-PUFAs tended to be higher (*P* = 0.053) after the experimental diet than after the reference diet (Table 5). The effect of both enteral diets on arachidonic acid (AA) and docosapentaenoic acid (DPA) were different in both groups. The administration of the experimental diet decreased AA after 90 days and increased DPA after 180 days. AA was unmodified and EPA decreased with the reference diet. Indeed, the reference diet increased palmitic acid at 90 days.

## 4. Discussion

The present study evaluated the effect of an* n*-3 LC-PUFA-enriched enteral formula on oxidative stress, the antioxidant defence system, inflammatory and CVD risk biomarkers, and the RBC fatty acid profile in fragile adults and elderly subjects to ascertain its suitability for nutritional treatment. The main results of the present study were that the enteral administration of* n*-3 LC-PUFAs tended to increase the RBC levels without an increase in LDL susceptibility to oxidation or DNA integrity. In addition, the plasma antioxidant capacity was maintained throughout the intervention. Reduced-CoQ_10_ and antioxidant enzymes (GPx, GR, and SOD) activities were decreased in the volunteers fed the* n*-3 LC-PUFA-enriched diet. Moreover, TAG and the CDV biomarker tPAI-1 decreased only after the intake of the* n*-3 LC-PUFA diet whereas MMP-9 decreased in both intervention groups. It is well documented that modest consumption of fish or fish oil supplementation positively affects cardiac haemodynamics [35], which could partially account for the clinical benefits of fish or fish oil intake, including a lower risk of cardiac death [36], cerebrovascular disease [37], ischaemic stroke, heart failure [38], cognitive decline [39], and nonfatal coronary events [40]. In addition, EPA and DHA-derived molecules exert anti-inflammatory and inflammatory-resolving effects (opposite of AA) and lower the production of inflammatory cytokines following reduced activation of proinflammatory transcription factors, such as NF-*κ*B [41, 42]. Therefore, increased* n*-3 LC-PUFAs in membranes may be helpful for a variety of acute and chronic inflammatory processes that occur during ageing [43]. On the other hand, a well-known beneficial effect of* n*-3 LC-PUFAs is the optimization of plasma TAG metabolism [44], a well-known cardiovascular risk biomarker. We have previously reported that feeding enteral formula enriched in EPA and DHA lowers plasma TAG without affecting insulin resistance [24]. In the present study, we have selected patients over 50 years old participating in that study [24] and we have also observed a significant effect on plasma TAG. No other plasma lipid was affected by the enteral feeding with any diet as previously reported [24].

However, the high degree of unsaturation of* n*-3 PUFA makes these fatty acids highly susceptible to oxidation, which may render them harmful, leading to dysfunctional membranes and to increased oxidative molecules that trigger inflammatory responses [4, 23]. In the present study, we observed that LDL particles were not more susceptible to* in vitro* oxidation after the administration of* n*-3 PUFAs. In addition, we did not observe increased* in vivo* plasma oxLDL levels or modifications on blood lymphocyte DNA oxidative damage. Indeed, serum TAC was also unmodified reflecting that the oxidative status was not impaired following the ingestion of* n*-3 LC-PUFAs for 6 months. Some authors have reported that* n*-3 LC-PUFA administration increased LDL susceptibility to oxidation [45, 46]. In accordance with our results, some authors have reported that fish oil supplementation does not increase the overall oxidation of LDL* ex vivo* [47] or the systemic oxidative stress [48, 49]. Furthermore, the increased susceptibility of LDL to* in vitro* oxidation observed in haemodialysis patients can be reduced by supplementation with fish oil containing vitamin E as an antioxidant [50]. Other authors have observed that supplementation of dairy products with* n*-3 LC-PUFA and an adequate amount of vitamin E decreases cardiovascular risk factors without causing additional oxidative DNA damage [51]. This may explain the oxidative stress stability of our volunteers fed the* n*-3 LC-PUFA-enriched diet, which is well stabilized against peroxidation (1 mg of *α*-tocoferol/g of* n*-3 PUFA, Table 1).

This oxidative stress stability may require lower antioxidant defences and that may be the reason for the lower induction of RBC antioxidant defence found in the present study. Although antioxidant enzyme activities decreased after* n*-3 LC-PUFA administration, this does not necessarily mean an impaired antioxidant capacity in RBCs but perhaps a compensation of activity in subjects with a lower risk of oxidative stress and with a frailty metabolism. The effect of* n*-3 LC-PUFA administration on RBC antioxidant enzymatic activity is not clear because some authors have described induction [52–55] and others have observed a null effect [49, 56, 57] depending on the administration dosage and duration. In addition, the decrease in plasma reduced-CoQ_10_ may be caused by a lower GR activity that is not able to regenerate from the oxidised form. On the other hand, we cannot explain the lower plasma concentrations of total and reduced CoQ_10_ after feeding the reference diet, but this fact may influence the higher rate of LDL oxidation found in this group of patients at the end of the intervention.

Proinflammatory molecules may be implicated in the initiation, progression, and plaque instability of the atherosclerotic process. MMPs are a large family of proteolytic enzymes involved in the remodelling of several components of the extracellular matrix that play a role in many physiological and pathological processes [58, 59]. The beneficial effect of* n*-3 LC-PUFAs lowering MMP-9 expression and activity has been previously reported [60–62], although other authors have reported no effect [63, 64]. Our data indicate that EN with both formulae reduced plasma MMP-9 levels. Therefore, we cannot attribute the beneficial effect to* n*-3 LC-PUFAs but to an adequate supply of nutrients because the reference product did not provide these ingredients and caused a similar effect.

We did not observe a positive effect of the* n*-3 LC-PUFA formula on plasma levels of any other inflammatory biomarker. The effects of* n*-3 LC-PUFAs are not clear. A recent meta-analysis provided consistent evidence that marine-derived* n*-3 PUFAs supplementation had a significant lowering effect on fasting blood levels of CRP, IL-6, and TNF-*α* in subjects with chronic nonautoimmune disease and healthy subjects. Those authors found a significant negative linear relationship between the duration and effect size of marine-derived* n*-3 PUFA supplementation on fasting blood levels of TNF-*α* and IL-6 [65]. In the present study, the plasma levels of hsCRP, IL-6, and TNF-*α* were relatively low at baseline, which could explain why* n*-3 LC-PUFAs had no observable effects later. With regard to CVD risk biomarkers, some studies report no influence on sICAM-1 after a high dose of* n*-3 LC-PUFAs [66, 67] but an increase in sVCAM-1 [67]. However, other studies have reported that* n*-3 LC-PUFAs may decrease these endothelial-derived molecules [68, 69]. In addition, Adkins and Kelley [70] reviewed the underlying cardioprotective mechanisms of* n*-3 LC-PUFAs, including the inhibition of monocyte infiltration due to decreased sVCAM-1, sICAM-1, and MCP-1 (another molecule that was not modified after* n*-3 LC-PUFAs feeding). In accordance with our results, some authors have described that* n*-3 LC-PUFA supplementation had no effect on the plasma levels of MPO in healthy adults [71].

Endothelin-1 is a potent vasoconstrictor peptide and accepted marker of increased oxidative stress secreted by endothelial cells. As previously described [72], we did not observe any effect of the inclusion of EPA and DHA on endothelin-1 in our patients. We observed that after 90 and 180 days of enteral feeding, the subjects receiving the* n*-3 LC PUFA-enriched formula had lower levels of circulating endothelin-1 than those in the reference group, which may be an indicator of a better vascular status. In addition, we identified differences between both groups for tPAI-1 plasma levels. The* n*-3 LC PUFA-enriched diet tended to diminish tPAI-1 plasma levels, and the reference diet did not cause this effect. tPAI-1 is associated with CVD risk and has an important role in the development of vascular thrombosis [73]. The beneficial effect of* n*-3 LC-PUFAs on tPAI levels has been previously reported [74]. We speculate that the frailty of patients included in the present study makes it difficult to find positive effects with the current doses and intervention times. Therefore, studies with longer intervention periods might be interesting to ascertain whether the formula has an impact on these vascular functional biomarkers, morbidity, and mortality.

In conclusion, the present work indicates that exclusive feeding with an enteral formula enriched with EPA and DHA does not impair the oxidative status of fragile adults and elderly patients and could have a positive effect on some inflammatory biomarkers demonstrating a nutritional tool that may help in the prevention of cardiovascular age-related complications without adverse effects.

## Figures and Tables

**Table 1:** Nutritional composition of the experimental and reference diets.

	Experimental	Reference
Energy (kcal/dL)	100	103
Proteins (g/dL)	4.0	4.0
Carbohydrates (g/dL)	12.3	14.05
Fat (g/dL)	3.9	3.47
Saturated (g/dL)	0.93	0.70
Monounsaturated (g/dL)	2.02	2.00
Polyunsaturated (g/dL)	0.95	0.77
Essential fatty acids (g/dL)	0.85	0.76
*n-*3 LC-PUFA (mg/dL)	110	—
Vitamin E (mg/L)	10	0.23
*n-*6 : *n-*3 ratio	6.3 : 1	10 : 1
Fibre (g/L)	1.70	1.44
Total minerals (g/dL)	0.71	0.57

The product contains a vitamin complex to satisfy 100% of the recommended vitamin intake for the elderly assuming a daily intake of 1500 kcal (6276 kJ). *n-*3 LC-PUFA: *n-*3 long chain polyunsaturated fatty acids.

**Table 2:** Plasma lipid profiles elderly patients were fed exclusively by total enteral nutrition with the experimental and reference diets for 90 d and 180 d.

	Experimental			Reference		
	Baseline (*n* = 25)	90 d (*n* = 19)	180 d (*n* = 11)	Baseline (*n* = 33)	90 d (*n* = 27)	180 d (*n* = 20)
TAG (mg/dL)^†^	164 ± 23	143 ± 17	140 ± 20	129 ± 10	117 ± 11	141 ± 22
TC (mg/dL)	170 ± 7	178 ± 9	189 ± 12	175 ± 8	162 ± 7	170 ± 11
LDLc (mg/dL)	97 ± 6	105 ± 8	110 ± 12	103 ± 6	95 ± 6	99 ± 10
ApoB (mg/dL)	80 ± 4	92 ± 6	85 ± 10	75 ± 4	75 ± 4	75 ± 7
HDLc (mg/dL)	47 ± 2	50 ± 3	54 ± 4	50 ± 3	49 ± 2	48 ± 3
ApoA-I (mg/dL)	133 ± 5	132 ± 6	135 ± 4	133 ± 4	131 ± 5	127 ± 4

^†^Significant differences per time, according to a general lineal model for repeated measures. *P* < 0.05 was considered significant. ApoA-I, apolipoprotein A-I; apoB, apolipoprotein B; TC, cholesterol; HDLc, high-density lipoprotein cholesterol; LDLc, low-density lipoprotein cholesterol; TAG, triacylglycerols; TC, total cholesterol.

**Table 3:** Blood oxidative stress parameters of frail adult and elderly patients fed exclusively by total enteral nutrition with the experimental and reference diets for 90 d and 180 d.

	Experimental			Reference		
	Baseline (*n* = 26)	90 d (*n* = 21)	180 d (*n* = 14)	Baseline (*n* = 28)	90 d (*n* = 24)	180 d (*n* = 19)
LDL susceptibility to oxidation						
Dienes lag Phase (min)	78.1 ± 3.4^b^	58.1 ± 3.0^a^	71.5 ± 4.0^b^	70.5 ± 3.0	67.67 ± 2.7	70.6 ± 4.6
Dienes slope (mU OD/min)	1.30 ± 0.09	1.56 ± 0.16	1.89 ± 0.24	1.26 ± 0.10^a^	1.28 ± 0.12^a^	1.82 ± 0.16^b^
LDL hydroperoxides^*∗*^	2.67 ± 0.21	3.66 ± 0.34	3.26 ± 0.18	4.56 ± 0.31	3.61 ± 0.39	4.15 ± 0.31
OxLDL (ng/mL)	167.7 ± 26.7	166.7 ± 25.2	175.6 ± 28.9	126.2 ± 12.5	156.5 ± 29.6	129.9 ± 14.1
DNA damage						
Comet head (%)	72.8 ± 2.2	76.8 ± 2.1	75.6 ± 1.6	75.45 ± 2.0	78.7 ± 1.7	79.8 ± 1.8
Comet tail (%)	24.4 ± 1.6	27.2 ± 2.2	23.2 ± 2.1	20.2 ± 1.8	24.5 ± 2.1	21.3 ± 1.7
Comet tail moment^*∗*^	0.53 ± 0.05	0.60 ± 0.08	0.51 ± 0.07	0.37 ± 0.05^&^	0.43 ± 0.05	0.37 ± 0.04

^*∗*^Significant difference between groups according to a general lineal model for repeated measures. ^&^Significant differences between groups at each time point. Mean values within a row with unlike superscript letters are significantly different in each group. *P* < 0.05 was considered significant. LDL hydroperoxide units are nmol/mg LDL protein. LDL, low-density lipoprotein; OD, optical density; oxLDL, oxidized low-density lipoprotein.

**Table 4:** Plasma cardiovascular and inflammatory biomarkers of frail adult and elderly patients fed exclusively by enteral nutrition with the experimental and reference diets for 90 d and 180 d.

	Experimental			Reference		
	Baseline (*n* = 26)	90 d (*n* = 21)	180 d (*n* = 14)	Baseline (*n* = 28)	90 d (*n* = 24)	180 d (*n* = 19)
MMP-9 (ng/mL)	156.6 ± 20.5^b^	112.6 ± 17.7^ab^	69.7 ± 8.6^a^	121.1 ± 12.9^b^	81.4 ± 13.4^a^	61.0 ± 8.6^a^
sVCAM-1 (*μ*g/mL)	1.07 ± 0.05	1.13 ± 0.08	1.34 ± 0.09	1.07 ± 0.08	1.08 ± 0.09	1.06 ± 0.10
sICAM-1 (ng/mL)	211.6 ± 12.6	214.3 ± 12.8	253.9 ± 23.0	199.6 ± 9.59	204.0 ± 11.1	189.6 ± 7.8
sE-Selectin (ng/mL)	16.59 ± 1.4	16.8 ± 1.3	17.4 ± 3.3	16.23 ± 1.2	15.6 ± 1.8	16.6 ± 1.7
MCP-1 (pg/mL)	202.0 ± 16.2	174.8 ± 16.9	161.6 ± 16.5	173.5 ± 13.4	144.6 ± 8.8	152.5 ± 20.5
Endothelin-1 (pmol/L)	2.22 ± 0.90	1.66 ± 0.94	1.80 ± 1.09	2.07 ± 0.50	1.84 ± 0.45^&^	2.32 ± 0.95^&^
tPAI-1 (ng/mL)^*γ*^	10.32 ± 2.26	8.76 ± 2.49	5.08 ± 1.41	7.44 ± 1.98	8.01 ± 2.30	8.63 ± 2.05
MPO (ng/mL)	21.76 ± 4.1	17.2 ± 3.0	14.3 ± 1.7	16.1 ± 2.1	16.7 ± 3.9	21.3 ± 3.8
TNF*α* (ng/mL)	7.06 ± 0.92	7.29 ± 1.69	6.52 ± 0.79	5.60 ± 0.61	4.87 ± 0.55	4.72 ± 0.55
IL-6 (pg/mL)	35.1 ± 6.5	40.0 ± 8.6	20.6 ± 7.5	35.8 ± 11.7	54.7 ± 18.6	40.2 ± 19.4
hsCRP (mg/L)	2.03 ± 0.42	2.12 ± 0.65	0.98 ± 0.32	2.02 ± 0.59	1.88 ± 0.60	1.88 ± 0.55

^&^Significant differences between groups at each time point. ^*γ*^Significant differences time per group according to a general lineal model for repeated measures. Mean values within a row with unlike superscript letters are significantly different in each group. *P* < 0.05 was considered significant. IL-6, interleukin*-*6; MCP-1, monocyte chemoattractant protein*-*1; MMP-9, metalloproteinase-9; MPO, myeloperoxidase; sICAM-1, soluble intercellular adhesion molecule-1; sVCAM-1, soluble vascular cell adhesion molecule-1; TNF-*α*, tumour necrosis factor-*α*; tPAI-1, total plasminogen activator inhibitor-1; hsCRP, high sensitive C reactive protein.

**Table 5:** Fatty acids in red blood cell membranes of frail adult and elderly patients fed exclusively by total enteral nutrition with the experimental and reference diets for 90 d and 180 d.

(%)	Experimental			Reference		
	Baseline (*n* = 25)	90 d (*n* = 21)	180 d (*n* = 14)	Baseline (*n* = 27)	90 d (*n* = 24)	180 d (*n* = 18)
Palmitic acid	23.3 ± 0.4	24.6 ± 0.4	23.6 ± 0.5	22.3 ± 0.8^a^	23.5 ± 0.3^b^	23.7 ± 0.3^ab^
Stearic acid	13.6 ± 0.4	13.6 ± 0.3	13.9 ± 0.2	13.6 ± 0.3	13.4 ± 0.3	13.8 ± 0.4
Oleic acid	17.6 ± 0.5	17.8 ± 0.5	17.5 ± 0.4	18.1 ± 0.4	17.9 ± 0.4	18.2 ± 0.6
LA^**∗**^	8.8 ± 0.3	9.6 ± 0.4	9.4 ± 0.4	8.6 ± 0.3	8.2 ± 0.3^&^	8.2 ± 0.2^&^
LNA^**∗**^	0.24 ± 0.01	0.22 ± 0.01	0.26 ± 0.02	0.3 ± 0.02^&^	0.29 ± 0.02^&^	0.27 ± 0.01
AA	14.0 ± 0.4^b^	12.3 ± 0.5^a^	12.3 ± 0.6^ab^	14.6 ± 0.4	13.5 ± 0.5	13.7 ± 0.4
EPA	0.59 ± 0.04	0.55 ± 0.04	0.58 ± 0.03	0.71 ± 0.05^b^	0.53 ± 0.04^a^	0.49 ± 0.04^a*γ*^
DPA^*γ*^	1.5 ± 0.1^a^	1.7 ± 0.1^a^	2.2 ± 0.1^b^	1.9 ± 0.4	1.8 ± 0.1	1.7 ± 0.1
DHA	4.0 ± 0.3	3.6 ± 0.2	3.6 ± 0.2	3.4 ± 0.3	2.8 ± 0.2^*γ*^	3.0 ± 0.2
*n-*3 LC-PUFA	6.0 ± 0.3	5.8 ± 0.3	6.4 ± 0.2	6.0 ± 0.5	5.2 ± 0.21	5.1 ± 0.3

^*∗*^Significant difference between groups according to a general lineal model for repeated measures. ^*γ*^Significant differences between groups at each time point. Mean values within a row with unlike superscript letters are significantly different in each group. ^&^Significant differences between groups at each time point. *P* < 0.05 was considered significant. AA, arachidonic acid; DHA, docosahexaenoic acid; DPA, docosapentaenoic acid; EPA, eicosapentaenoic acid; LA, linoleic acid; LNA linolenic acid; *n*-3 LC-PUFA, omega-3 long chain polyunsaturated fatty acids.
